# Human Bone Marrow Mesenchymal Stem Cells Induce Collagen Production and Tongue Cancer Invasion

**DOI:** 10.1371/journal.pone.0077692

**Published:** 2013-10-21

**Authors:** Sirpa Salo, Carolina Bitu, Kalle Merkku, Pia Nyberg, Ibrahim O. Bello, Jussi Vuoristo, Meeri Sutinen, Hannu Vähänikkilä, Daniela E. Costea, Joonas Kauppila, Petri Lehenkari, Dan Dayan, Marilena Vered, Juha Risteli, Tuula Salo

**Affiliations:** 1 Department of Diagnostics and Oral Medicine, Institute of Dentistry, University of Oulu, Oulu, Finland; 2 Department of Oral Medicine and Diagnostic Oral Sciences, King Saud University, College of Dentistry, Riyadh, Saudi Arabia; 3 Department of Pedodontics, Cardiology and Endodontology, Institute of Dentistry, University of Oulu, Oulu, Finland; 4 Section for Pathology, the Gade Institute University of Bergen Haukeland Hospital, Bergen, Norway; 5 Department of Pathology and Department of Surgery, University of Oulu, , and Oulu University Hospital, Oulu, Finland; 6 Department of Anatomy, University of Oulu, Oulu, Finland; 7 Department of Oral Pathology and Oral Medicine, School of Dentistry, Tel Aviv University, Tel Aviv, Israel; 8 Institute of Pathology, the Chaim Sheba Medical Center, Tel Hashomer, Israel; 9 Institute of Diagnostics, Department of Clinical Chemistry, University of Oulu, and NordLab, Oulu University Hospital, Oulu, Finland.; 10 Institute of Dentistry, University of Helsinki, Helsinki, Finland; 11 Oulu University Hospital, Oulu, Finland; 12 Medical Research Center, Oulu, Finland; University of Sao Paulo - USP, Brazil

## Abstract

Tumor microenvironment (TME) is an active player in carcinogenesis and changes in its composition modify cancer growth. Carcinoma-associated fibroblasts, bone marrow-derived multipotent mesenchymal stem cells (BMMSCs), and inflammatory cells can all affect the composition of TME leading to changes in proliferation, invasion and metastasis formation of carcinoma cells. In this study, we confirmed an interaction between BMMSCs and oral tongue squamous cell carcinoma (OTSCC) cells by analyzing the invasion progression and gene expression pattern. In a 3-dimensional myoma organotypic invasion model the presence of BMMSCs inhibited the proliferation but increased the invasion of OTSCC cells. Furthermore, the signals originating from OTSCC cells up-regulated the expression of inflammatory chemokines by BMMSCs, whereas BMMSC products induced the expression of known invasion linked molecules by carcinoma cells. Particularly, after the cell-cell interactions, the chemokine CCL5 was abundantly secreted from BMMSCs and a function blocking antibody against CCL5 inhibited BMMSC enhanced cancer invasion area. However, CCL5 blocking antibody did not inhibit the depth of invasion. Additionally, after exposure to BMMSCs, the expression of type I collagen mRNA in OTSCC cells was markedly up-regulated. Interestingly, also high expression of type I collagen N-terminal propeptide (PINP) *in vivo* correlated with the cancer-specific mortality of OTSCC patients, whereas there was no association between cancer tissue CCL5 levels and the clinical parameters. In conclusion, our results suggest that the interaction between BMMSC and carcinoma cells induce cytokine and matrix molecule expression, of which high level of type I collagen production correlates with the prognosis of OTSCC patients.

## Introduction

The tumor microenvironment (TME) undergoes extensive changes during tumor growth [1] and the progression of a tumor is dependent on stromal elements [2]. Cells in the microenvironment, including carcinoma-associated fibroblasts (CAFs), bone marrow-derived multipotent mesenchymal stromal cells (BMMSCs), tumor associated macrophages (TAMs) and other inflammatory cells as well as vascular cells all contribute to varying degrees to the hallmarks of cancer and cancer ecosystem [3] [4]. They produce extracellular matrix, growth factors, cytokines, proteases and their regulators, and thus, provide a microenvironment supporting cancer cell proliferation and immortality, inducing angiogenesis, reprogramming energy metabolism, evading immune destruction, and favoring invasion and metastasis [5],[1,3,6], [4]. 

In tongue cancer the components of TME have an elementary role in the invasion and metastasis processes with a direct impact on patients’ clinical outcomes [7]. We have shown that the high frequency of CAFs is associated with poor prognosis in mobile tongue cancer patients [8], [9]. CAFs have also been shown to localize at the site of metastatic lymph node similarly to matched primary tongue tumors suggesting facilitation of metastasis [10]. Our recent study profiled the molecular cross-talk between oral cancer cells and TME and presented that the examination of known pro-tumorigenic components of the inflammatory infiltrate, such as regulatory T cells, TAM2 (i.e. TAM subtype supporting invasion and metastasis) cells, and regulatory T-cell inducing immune cells, revealed negative impact for patients similar to CAFs [11]. 

BMMSCs have been shown to incorporate into damaged or inflamed tissue as well as to home at tumors and the site of metastasis where they integrate into the TEM and provide a source for cells, such as CAFs [12], [13] [14], [15],, [2]. Cytokines and growth factors secreted by tumor cells together with endocrine factors of inflammatory tissues surrounding tumors attract BMMSCs to tumor stroma [16]. BMMSCs have been shown to promote invasion and metastasis in various cancers, such as breast, colon and lymphatic cancers [17], [18], [19]. However, the impact and the role of BMMSCs in TEM and the mechanisms of their potential effects on different tumors still remain controversial [20], [21]. 

In addition to various cell types, the extracellular matrix (ECM) proteins in TME can also act as crucial factors in dynamic informational system influencing cancer outcome [22]. The most abundant protein in TME is type I collagen which leads to the tumor growth, invasion and spreading of cancer. Particularly, the release of the aminoterminal propeptide of type I procollagen (PINP) indicates the tumor-induced fibro-proliferative response [22-24].

The objective of this work was to investigate the effect of the BMMSCs and carcinoma cells interactions on OTSCC gene expression, invasion and clinical outcome of the OTSCC patients. Here we demonstrated that BMMSCs induced OTSCC carcinoma cell invasion *in vitro* partially through chemokine CCL5 signaling since its inhibition reduced the invasion area. In OTSCC cells the expression of type I collagen mRNA was up-regulated by signals derived from BMSCC, and the high expression level of immunoreactive type I procollagen correlated with the cancer-specific mortality of the OTSCC patients. 

## Materials and Methods

### Cell culture

Human tongue squamous cell carcinoma cells HSC-3 (JCRB 0623; Osaka National Institute of Health Sciences, Osaka, Japan), SAS (JCRB 0260; Osaka National Institute of Health Sciences, Osaka, Japan) and human dysplastic oral keratinocytes DOK (European Collection of Cell Cultures 94122104, Salisbury, Wilts., UK) were cultured in 1:1 DMEM/F-12 (Invitrogen) supplemented with 100 U/ml penicillin, 100 µg/ml streptomycin, 50 µg/ml ascorbic acid, 250 ng/ml fungizone, 5 µg/ml insulin (bovine pancreas), 0.4 µg/ml hydrocortisone (all from Sigma-Aldrich), and 10% heat-inactivated fetal bovine serum (FBS). For zymography, fetal bovine serum was replaced by 0.5% lactalbumin (Sigma-Aldrich). Human bone marrow-derived BMMSCs were originally obtained from patients operated for hip fracture or osteoarthritis. The ethical committee of Oulu University Hospital had approved the study protocol (Statements 4/2000,58/2009 and 21/2011; Research Diary 180/2001 and 12/2004) and the patients had given their informed written consent for participation in the study. The BMMSCs used in this study were harvested and cultured as described previously [25][26]. All experiments were carried out with cells with low passage numbers 3 - 4. Human gingival fibroblasts (GF) used in this study were obtained from biopsies of healthy gingiva as described earlier [27]. Carcinoma-associated fibroblasts (CaDEC12) [11]derived from a specimen of tongue SCC as well as normal oral fibroblasts (NOFs) [28] were cultured in the same media as GFs [27]. All cells were cultured in a humidified atmosphere of 5% CO_2_ at 37°C. In BMMSC or GF co-cultures with HSC-3, SAS or DOK cells BMMSC or GF culture media was used. 

### Organotypic invasion assay

The organotypic invasion assay and the quantification of invasion were performed as described previously [29]. In monoculture assays 2.0 x 10^5^ - 4 x 10^5^ HSC-3 or SAS cells or 2 x 10^5^ DOK cells were cultured on the top of myoma disk for 10 - 14 days. In co-culture assays 0.5 - 1.5 x 10^5^ BMMSCs were added together with cancer cells on the top of the myoma disks (OTSCC:BMMSC ratio varied from 2:1 to 5:1). The histological sections of myoma disks were stained with monoclonal pancytokeratin antibody (DAKO, clone AE1/AE3). The average of invasion area or depth of HSC-3, SAS, DOK or any other control cells grown in monoculture was set as 100 %. For the inhibition of invasion, 50 µg/ml of monoclonal antibody against human CCL5 (R&D Systems, MAB678), CXCL1 (R&D Systems, MAB275) or isotype matched mouse normal IgG (Jackson Laboratories) were added to cell culture media. 

### Proliferation assays

Quantification of proliferation in the organotypic invasion assay was performed from triplicate myoma disks per cell line used. Histological sections cut from the disks representing inner parts of the disk were immunostained with polyclonal antibody against Ki67 (Abcam, #15580). Cell proliferation rate was determined as the percentage of Ki67-expressing cells among all cells per microscopic field in the cell layer on the top of the myoma disk. Cancer cells were first labeled with Vybrant® CM-DiI cell-tracking solution (Life Technologies) for the discrimination of cancer cells from BMMSCs. Altogether four microscopic fields were counted per section (12 fields per sample). For the cell culture proliferation assay 2 x 10^4^ HSC-3 cells were cultured as a monoculture or as a co-culture with 1 x 10^4^ BMMSCs or GFs on four chamber slides (Lab-Tek) per test for 24 h. The slides were stained with polyclonal antibody against Ki67 and Alexa Fluor® 488 secondary antibody (Molecular Probes) and counterstained with DAPI. 10 microscopic fields per chamber slide were examined and cell proliferation rate was determined as a percentage of Ki67-expressing cells among all cells per microscopic field. 

### Scratch assay

A total of 1 x 10^5^ HSC-3 cells and 1 x 10^5^ BMMSCs or GFs were co-cultured in 10% FCS complete culture media in 24-well plates (Costar) in triplicate wells until confluence after which a wound was made by using a pipet tip. The wells were washed with cell culture media and the wounded areas were photographed immediately after wounding (0 h) and again in the end of the study (20 h) when cells were stained with crystal violet. The size of the wound area and the closure of wound were analyzed with ImageJ (version 1.45s, http://imagej.nih.gov/ij/). The completely healed wound area was set to a value 100. 

### Zymography

Zymography performed as previously described [29] was used to detect the expression of metalloproteinases 2 and 9 (MMP-2 and -9) in HSC-3, SAS and DOK cell culture media after o/n incubation with 100 ng/ml of recombinant CCL5 (rCCL5) (R&D Systems, #278-RN). 

### Microarray

HSC-3 cells were cultured as a monoculture or in a co-culture with BMMSC cells (cell ratio 1:1) on 6-well plates in two separate experiments for 24 h. Next HSC-3 cells were sorted out from co-cultures with FACS (FACScan, Becton Dickinson). RNA was extracted and purified from the cells with the Qiagen RNeasy kit according to the manufacturer's instructions and pooled for microarray. 

In another set of co-culture assay, 80% confluent cultures of HSC-3 were grown in HSC-3 media with 2% FBS for 24 h. The media were collected, centrifuged, transferred to BMMSC cultures and incubated for 24 h, after which the cells were collected. RNA was extracted as described above from three separate co-culture sets. 

Affymetrix Human GeneChip Arrays were used for Microarray analysis. Experimental procedures for GeneChip were performed according to the Affymetrix GeneChip Expression Analysis Technical Manual. The expression data was analyzed using the Affymetrix GeneChip Operating System (Affymetrix) and dChip software [30]. The array data were also deposited in the GEO (accession number GSE44458).

### Determination of chemokine levels

For the measurement of CCL5 expression 2 x 10^4^ HSC-3, SAS and DOK cells were cultured as a monoculture or in co-culture with 2 x 10^4^ BMMSCs or GFs (cell ratio 1:1 to 2:1) on 24-well plates (Costar). Cell culture media was collected 40 h after cell plating and filtered. The expression of chemokine CCL5 was determined from cell culture media by Quantikine CCL5/RANTES Immunoassay (R&D Systems). 

### Patient samples and clinicopathological data

Archival specimens of 105 OTSCC and ten lymph node metastases (pN1) from patients, surgically treated at the Oulu University Hospital between the years 1981-2009, were retrieved from the Oulu University Hospital, Department of Pathology, Oulu, Finland. Additional nine patients signed out as tumor-free lymph nodes (pN0) were retrieved from The Chaim Sheba Medical Center, Tel Hashomer, Ramat Gan, Israel. The median age of 114 patients was 64 years (range 27-87). The median follow-up time was 101 months (range 18-298 months) in the surviving patients (n=53). The median follow-up time for the study patients was 47 months (range 1-298 months). Patient survival data was acquired from Statistics Finland and other relevant data from patient records (Table 1). We could not retrieve treatment data from three of the patients and survival data from two of the patients. The retrieval of the patient data was approved by the local ethics committees and Finnish National Supervisory Authority for Welfare and Health. 

**Table 1:** Patient clinical data.

		**N**	**%**
**Age at diagnosis**			
	<55 yrs	40	35.1
	55-70 yrs	30	26.3
	>70 yrs	44	38.6
**Sex**			
	Male	56	49.1
	Female	58	50.9
**Tumour grade**			
	1	40	35.1
	2	62	54.4
	3	12	10.5
**Tumour stage**			
	1-2	63	55.3
	3-4	51	44.7
**Neck lymph nodes**			
	Negative	79	69.3
	Positive	35	30.7
**Neck dissection**			
	No	16	14.0
	Yes	98	86.0
**Adjuvant therapy**			
	No	68	59.6
	Radiotherapy	34	29.8
	Radio- and chemotherapy	9	7.9
	Missing data	3	2.6
	
**Total**		114	

### Immunohistochemistry

Immunostaining was performed on all of our OTSCC samples, which were previously selected to be representative of the tumour mass in the resected specimens. After a high temperature antigen retrieval method with citrate buffer for CCL5 (REAL Target Retrieval Solution, pH 6; Dako, Glostrup, Denmark) or Tris/EDTA for PINP (10 mmol/L Tris, 1 mmol/L EDTA, pH 9), sections were blocked by incubation with normal goat serum for 30 min. Slides were incubated overnight at 4°C with the polyclonal goat anti-human CCL5 antibody (#AF-278-NA, R&D Systems) at a 1:100 dilution. For the polyclonal rabbit anti-human antibody PINP [31] slides were incubated for one hour at room temperature with the PINP primary antibody at a 1:5000 dilution. Samples were incubated with secondary antibodies specific for each species. For detection we used Dako EnVision kit (Dako) with Diaminobenzidine (Dako basic DAB-kit) as a chromogen. Counterstaining was done in the Dako Autostainer (Dako, Copenhagen, Denmark). In negative controls IgG of each respective species was used instead of primary antibody.

### Immunohistochemical assessment of PINP and CCL5 expression

Expression of PINP and CCL5 was evaluated through immunohistochemistry. Samples were analyzed by three independent researchers (K.M., J.H.K., and T.S. or S.S., J.H.K., and T.S.; lymph nodes free of metastatic disease were examined by D.D. and M.V.). Different patterns of classifying expression were chosen. At first samples were classified based on the overall difference of staining between the superficial and invasive areas of the tumors were categorized (staining appears more intensive in superficial areas of the tumor or staining appears more intensive in invasive areas of the tumor). The percentage of positive cells in the stromal and tumor cells in both the superficial and invasive areas of the tumor was scored on a four point scale (0 = 0%, 1 = 0-25%, 2 = 25-50% and 3 = >50%, titled no, low, moderate and high expression, accordingly). Additionally, the presence of PINP staining in blood and lymphatic vessels was assessed. The subepithelial stroma of morphologically normal epithelium was also stained. At dysplastic mucosa sites both epithelial cells and the subepithelial stromal cells were analyzed. 

### RNA Interference

Three commercial CCR5 short hairpin RNA (shRNA) oligonucleotides (Thermo Scientific #VGH5518-98903425, #VGH5518-99159618, #VGH5518-99294601) and non-silencing control (Thermo Scientific #RHS4348) were obtained from Thermo Scientific Open Biosystems GIPZ Lentiviral shRNAmir Library (Thermo Scientific). The transductions of HSC-3 cells with viral and control vectors were performed according to manufacturer’s instructions with puromycin (Sigma-Aldrich) selection. The efficiency of knockdown by CCR5-shRNA was determined by flow cytometric analysis using monoclonal anti-CCR5 antibody (BD Pharmingen, #556903) and isotype matched IgG (BD Pharmingen, #555576) as a control. About 70 % knockdown of CCR5 was obtained compared to non-silencing control cells with one of the commercial oligonucleotides (Thermo Scientific #VGH5518-99159618). 

### Statistical analysis

All assays were repeated 2 - 4 times, except the 3D invasion assay with SAS and DOK cells that was performed only once with triplicate myoma disks per mono- or co-culture. Differences in cell proliferation and wound healing, invasion area and depth were evaluated by using Student’s t-test. In all experiments, a *p*-value of less than 0.05 was considered statistically significant. For statistical analyses of the patient material we used PASW Statistics 18 (IBM corp.) software. A chi-square-test was used to calculate statistically significant differences between prognostic and clinicopathological variables. Survival tables were calculated according to the Kaplan-Meier method, and were compared with the log-rank test. Multivariate survival and recurrence analyses were done with the Cox proportional hazards model using the following covariates: gender (male or female), age at the time of diagnosis (<55 yrs, 55-70 yrs and >70 yrs), tumour stage (1–4) and tumour histologic grade (well =1, moderately = 2 and poorly = 3 differentiated carcinomas according to World Health Organization head and neck carcinoma classification (2005) together with PINP expression pattern variables as indicated. Cox regression was done using backward stepwise selection of variables, and a *p* value of 0.05 was adopted as the limit for inclusion of a covariate. 

## Results

### BMMSCs induce tongue cancer cell invasion in the 3D in vitro organotypic model

**Figure 1:**
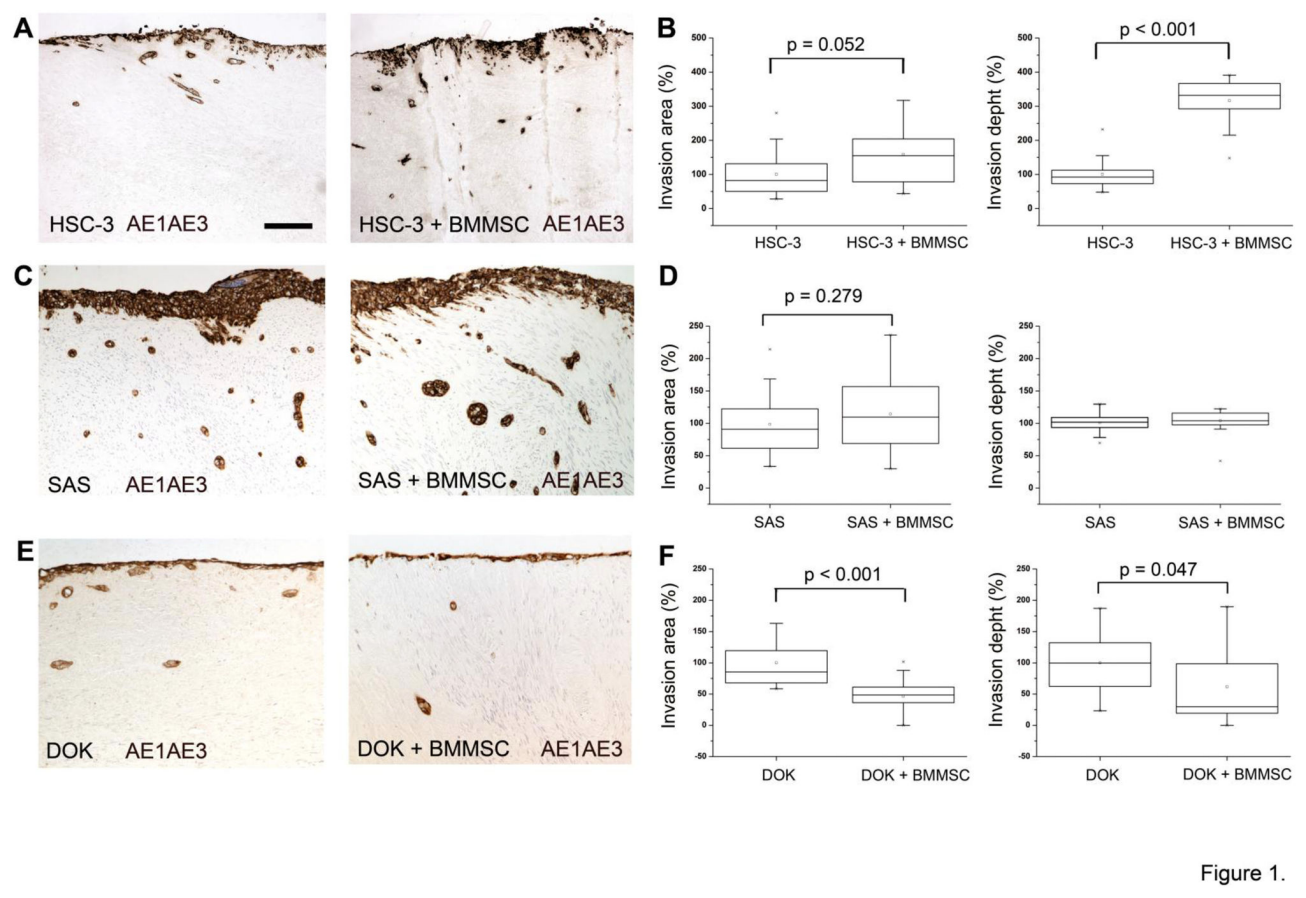
BMMSCs enhance tongue cancer cell invasion in 3D organotypic model. Bone marrow-derived BMMSCs enhance the invasion of HSC-3 cells (A, B), but not dysplastic cells (E, F) in 3D myoma organotypic model [29]. Minor increase, although not statistically significant, was seen in the invasion area of SAS (C,D). Histological sections obtained from myoma disks were stained with monoclonal pancytokeratin antibody (DAKO, clone AE1/AE3) and photographed at 100 x magnifications with a DMRB photo microscope connected to DFC 480 camera using QWin V3 software (Leica Microsystems). Scale bar 100 µm.

The effect of BMMSCs on tongue cancer cell invasion was examined in the myoma organotypic model [29] that is more faithful to the human TME than previous animal tissue derived models. Human tongue carcinoma cells, HSC-3, SAS or dysplastic DOK cells were cultured as a monoculture or in co-culture with BMMSCs on the top of myoma disks. After 10 - 14 days invasion area and depth were quantitated by analyzing images taken from pancytokeratin stained histological sections from monoculture and co-culture disks. As shown in Figure 1, BMMSCs induced an increase in invasion area, and even a clearer effect was seen on the invasion depth in HSC-3-BMMSCs co-cultures (Figure 1A-1B). A slight increase in the invasion area was seen also with SAS cells in the presence of BMMSCs, although not statistically significant. However, there seemed to be no effect on invasion depth (Figure 1C-1D). Interestingly, BMMSCs inhibited the invasion of dysplastic DOK cells (Figure 1D-1E) suggesting a different effect of BMMSCs on carcinoma cells compared to non-malignant cells. 

**Figure 2:**
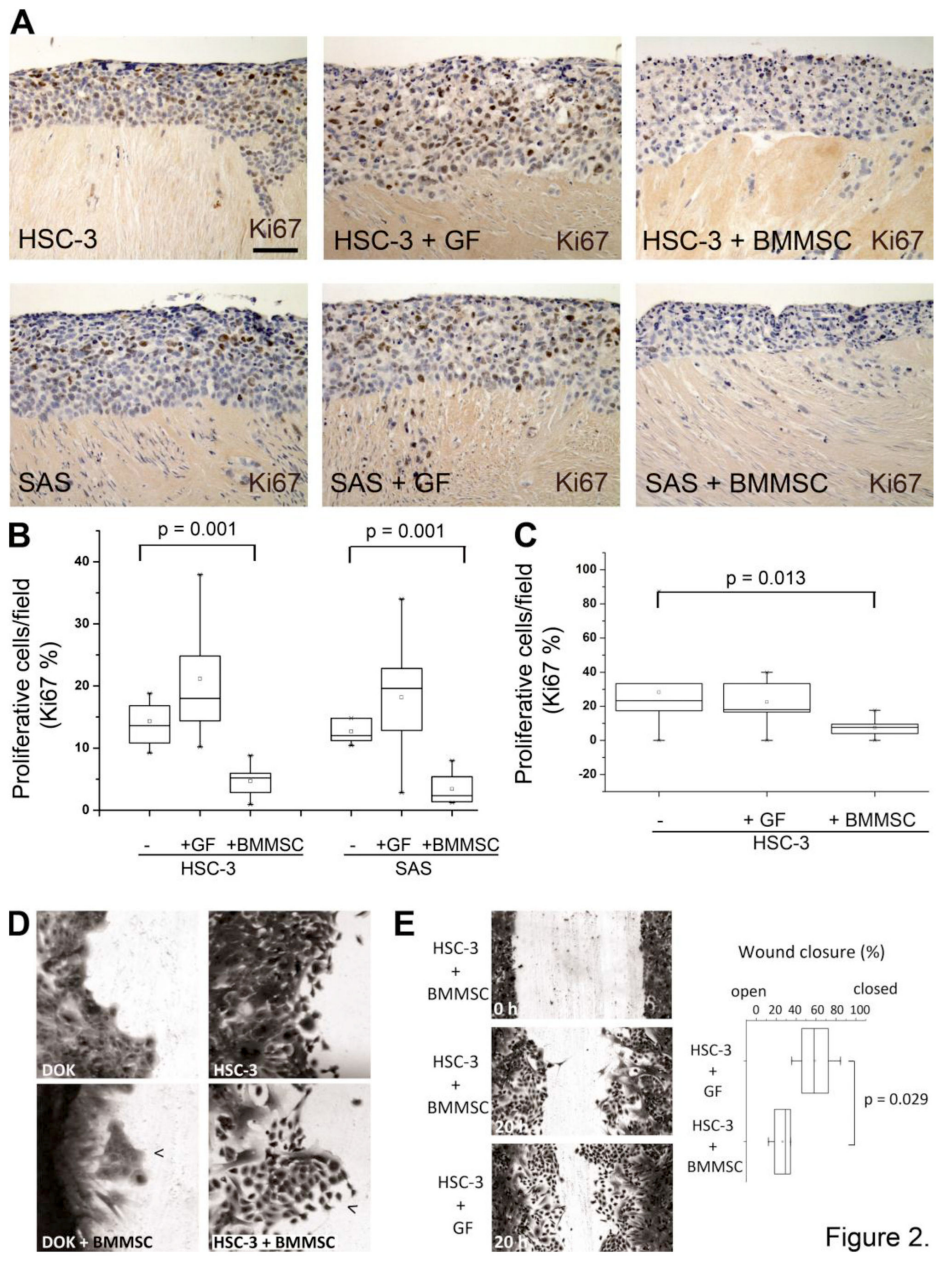
BMMSCs inhibit cell proliferation in tongue cancer cells. Histological sections obtained from myoma disks were stained with polyclonal anti-Ki67 antibody. BMMSCs inhibit proliferation of HSC-3 and SAS cells in organotypic invasion model (A, B) as well as in cell culture assays (C), and shift HSC-3, but not dysplastic DOK cells towards a migratory phenotype with less cell-cell contacts (D, arrowheads). Normal gingival fibroblasts, GFs, promote wound closure more than BMMSCs potentially partly due to the increase in cell proliferation of cancer cells (E). Scale bar 50 µm.

To study if the increased invasion of HSC-3 cells was due to increased cell proliferation, HSC-3 and SAS cells were cultured on top of myoma disk as a monoculture or co-culture with BMMSCs or GFs. Histological sections obtained from these disks were immunostained with the proliferation marker Ki67 and positive cells were calculated as described in Materials and Methods. Cell proliferation levels were remarkably lower in HSC-3 and SAS co-cultures with BMMSCs as compared to co-cultures with GFs (Figure 2A-2B). To confirm that the effect seen in histological sections came from the reduced proliferation of cancer cells, HSC-3 cells were cultured as a mono- or co-culture on cell culture chamber slides with BMMSCs labeled with a fluorescent dye. After co-culture overnight, cells were stained for Ki67. As shown in Figure 2C, BMMSCs inhibited HSC-3 cell proliferation significantly also in the cell co-culture assay. The viability of HSC cells remained still high and no sign of apoptosis was detected (data not shown). 

As proliferation was not increased, to evaluate if the increased invasion was a consequence of a higher cancer cell migration a scratch assay was performed with HSC-3 and DOK cells seeded alone or together with BMMSCs or GFs to 24-well plates. BMMSCs shifted HSC-3 cells, but not dysplastic DOK cells, towards a migratory phenotype with lamellipodia structures and less cell-cell contacts (Figure 2D). However, GFs promoted faster wound closure than BMMSCs, which may be partly due to effects of serum on the proliferation capacity of cancer cells (Figure 2D-2E)

### The expression of chemokine CCL5 is up-regulated in BMMSCs after interaction with cancer cells

**Figure 3:**
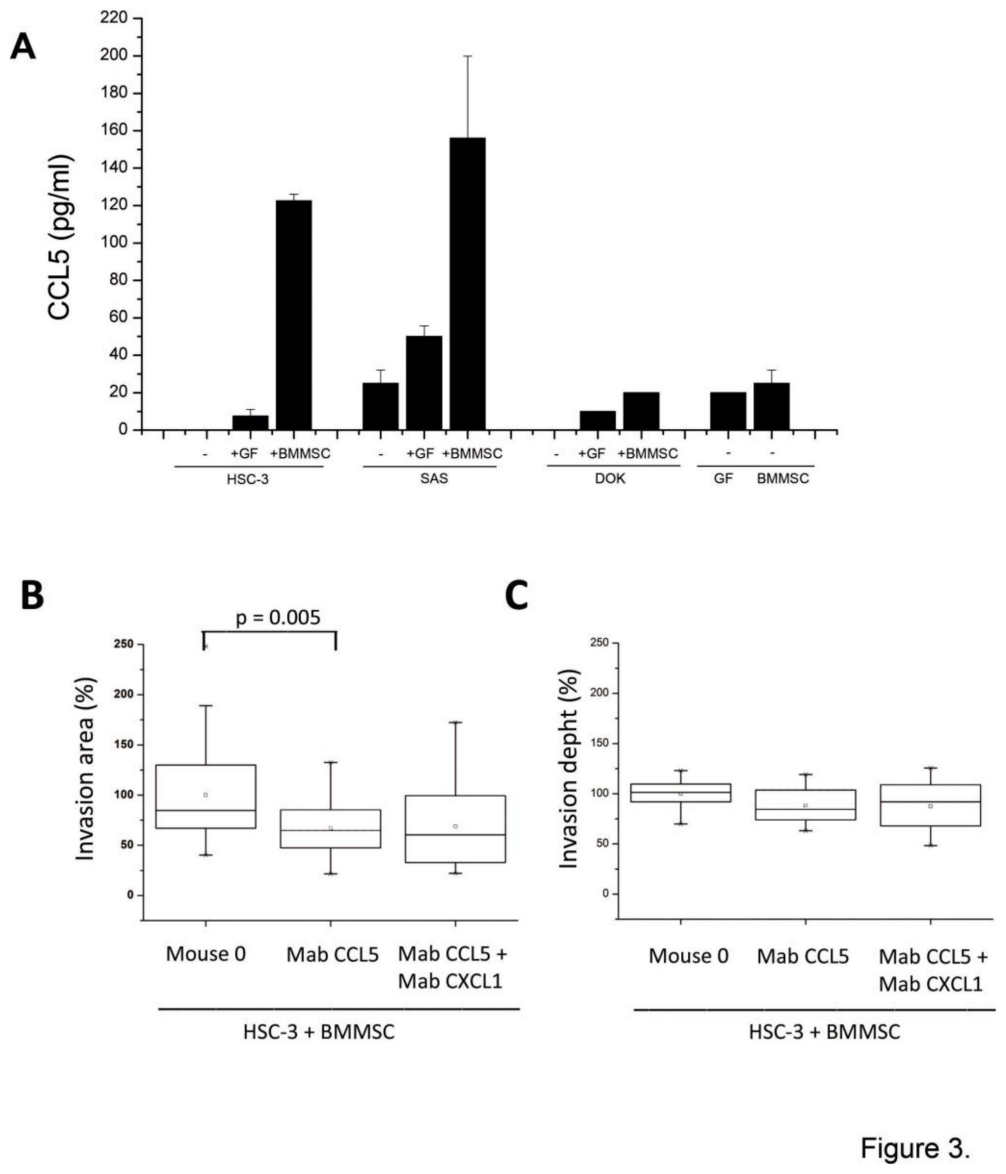
The expression of chemokine CCL5 is up-regulated in co-cultures of BMMSCs cells and OTSCC. Co-culture of OTSCC cells with BMMSCs results in the high expression of chemokine CCL5 in HSC-3 and SAS cells, but not in dysplastic DOK cells (A). Function-blocking monoclonal antibody against CCL5 (Mab CCL5) slightly inhibits BMMSC enhanced invasion area, but has no effect on the invasion depth, whereas the antibody against CXCL1 does not increase the inhibitory effect in OTSCC invasion (B, C).

BMMSCs were then cultured with conditioned media obtained from HSC-3. Although several genes were up-regulated, the highest increase in gene expression in BMMSCs was seen in chemokine genes (Table 2). When the chemokine expression was examined at protein level the expression of CCL5 was found to be primarily a result of the interaction between HSC-3 and BMMSC cells rather than from the co-cultures of HSC-3 cells and GFs (Figure 3A). Similar findings were obtained from co-cultures of SAS and BMMSCs, but not from DOK cells interacting with BMMSCs (Figure 3A). At protein level, the up-regulation of most of the chemokines listed on the Table 2 was found to originate from OTSCC cell lines interactions with both BMMSCs and GFs. Some chemokines, such as CCL2, CCL20 and CXCL8, were also up-regulated as a result of interactions between BMMSCs and DOK cells, as studied in immunoassays (data not shown).

**Table 2:** Up-regulated chemokine genes in BMMSCs after exposing to conditioned HSC-3 cell culture media (fold change > 2).

**Gene**	**Symbol**	**Fold Change**
chemokine (C-C motif) ligand 2	CCL2	830.66
chemokine (C-C motif) ligand 5	CCL5	194.90
chemokine (C-C motif) ligand 20	CCL20	182.23
chemokine (C-X-C motif) ligand 1	CXCL1	134.91
chemokine (C-X-C motif) ligand 2	CXCL2	80.98
chemokine (C-X-C motif) ligand 3	CXCL3	80.14
chemokine (C-X-C motif) ligand 5	CXCL5	73.90
chemokine (C-X-C motif) ligand 8 (interleukin 8)	CXCL8 (IL8)	18.06
chemokine (C-C motif) ligand 13	CXCL13	16.86

### Function-blocking antibody against CCL5 inhibits BMMSC increased invasion area

We next explored the importance of CCL5 in the spread of tongue cancer, since CCL5 has been shown to promote the motility of oral cancer cells [32]. CCL5 was also shown to be important in tumor progression of several cancers, such as breast and colorectal carcinoma [17,33]. Therefore, we tested the effect of CCL5 function-blocking antibody on OTSCC and BMMSC co-cultures in the 3D invasion model. We also tested the potential effect of function blocking antibody against CXCL1 on invasion, since CXCL1 was also expressed from BMMSC-HSC-3 interaction and it has previously been demonstrated to be present in cancers including breast, lung, colorectal and prostate cancers either supporting or suppressing tumor progression [34–36]. In oral cancer CXCL1 has been suggested to have a role in tumor progression since in patient samples the expression of CXCL1 has been shown be associated with leukocyte infiltration, lymph node metastasis, and angiogenesis [37]. As shown in Figure 3, the CCL5 function–blocking monoclonal antibody was able to significantly attenuate BMMSC-promoted HSC-3 cell invasion area, but had no effect on invasion depth (Figure 3B-3C). CXCL1 did not have an effect on invasion as it did not increase the inhibitory effect of CCL5 in the 3D invasion model (Figure 3B-3C). 

### In patient samples CCL5 is expressed mainly by inflammatory cells and some cancer cells, but only sparse CAFs are CCL5 positive

**Figure 4:**
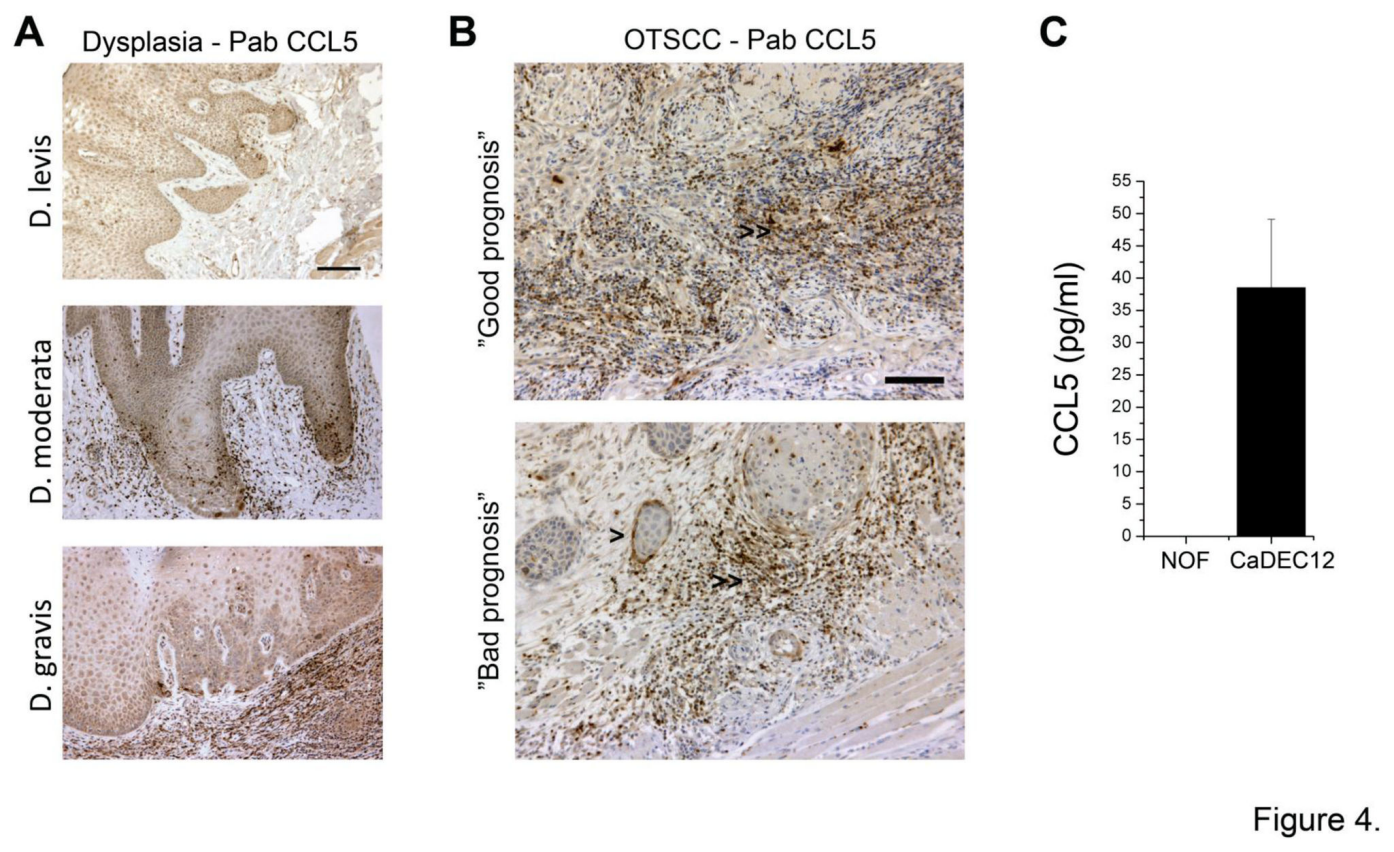
CCL5 expression in OTSCC patient samples. CCL5 is mainly expressed by inflammatory cells in TME and some cancer cells in patient samples as shown by immunostaining with Pab CCL5, but only sparse CAFs are positive for CCL5 (A, B). CaDEC12 cells, the primary cultures of CAFs obtained from tongue cancer patients, express relative amount of CCL5 compared to NOFs, normal oral fibroblasts (C). Scale bar 100 µm.

Next, to evaluate the importance of CCL5 expression in patient tumor samples for diagnostics of tongue cancer, histological sections representing different stages of dysplastic lesions and tongue carcinomas from human patients were stained with anti-CCL5 antibody. The expression of CCL5 was mostly detected in inflammatory cells and in some cancer cells (Figure 4A-4B), but only sparse CAFs were CCL5 positive, as assessed by immunohistochemical co-localization of CCL5 and CAF marker αSMA (data not shown). However, the expression of CCL5 was not associated with the clinical outcome of the OTSCC patients (not shown). The expression of CCL5 was further studied in normal primary oral fibroblasts (NOF, [28]) and CaDEC12 cells [11]. Immunoassay detecting secreted CCL5 in cell culture media showed very low or no CCL5 expression in normal fibroblasts, but considerable higher expression in CaDEC12 cells (Figure 4C). 

### The role of CCL5/CCR5 axis is not critical in BMMSC increased tongue cancer invasion

**Figure 5:**
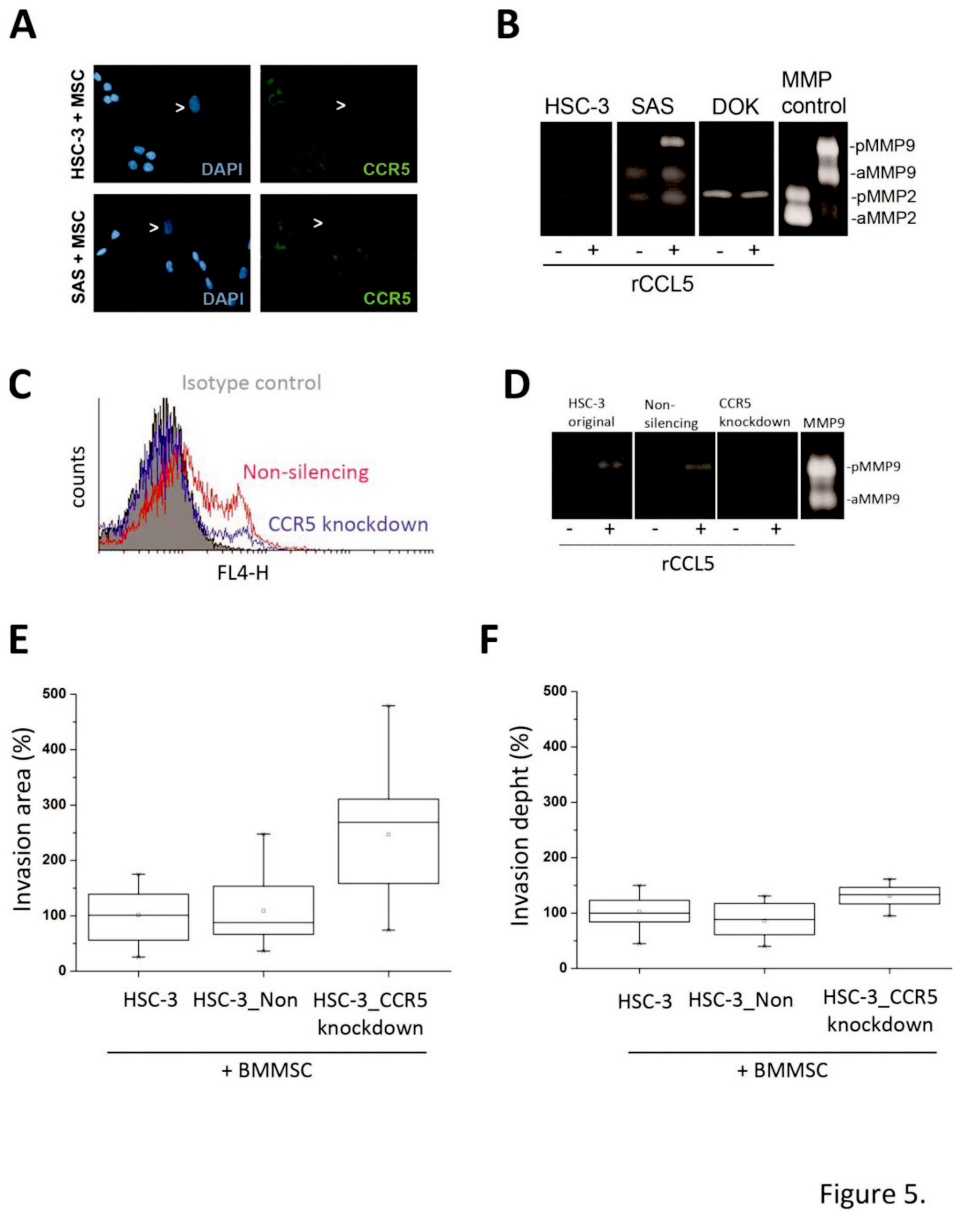
Signaling via CCL5/CCR5 is not critical in OTSCC invasion. The CCL5 receptor CCR5 is expressed in HSC-3 and SAS cells, but not in BMMSCs (A). rCCL5 can induce the expression of MMP9 in HSC-3 and SAS, but not in DOK cells (B). The silencing of the expression of CCR5 receptor in HSC-3 cells downregulates the gene expression about 70 % compared to control cells and blocks the signaling increasing the expression of MMP9 in cancer cells (C, D). CCR5 knockdown have minor or no effect on BMMSC enhanced invasion of HSC-3 cells in 3D organotypic model (E, F).

The CCL5 signaling in cancer cells has been proposed to be mediated mainly, but not necessary exclusively, via CCR5 receptor [38]. Both HSC-3 and SAS cells were shown to express this receptor (Figure 5A). In co-culture roughly all cancer cells expressed CCR5, however, in monocultures approximately only 25 % of cancer cells were positive for CCR5 suggesting that a contact with stromal cells or CCL5 induction is needed for higher CCR5 expression (flow cytometry analysis, data not shown). The association of CCL5 and CCR5 expression has been proposed recently also by others [39,40]. As the CCL5-CCR5 axis has been shown to promote the motility of human oral cancer cells *in vitro* [32] and to induce the expression of MMP9 [32], we also examined the effect of rCCL5 on MMP9 expression levels in HSC-3 and SAS as well as in dysplastic DOK cells. Similarly to previous observation by Chung and co-workers [32] rCCL5 clearly increased the expression of MMP9 in HSC-3 cells and SAS cells, but not in dysplastic DOK cells (Figure 5B). However, rCCL5 did not induce epithelial-mesenchymal transition (EMT) as analyzed by Western blotting with anti-vimentin antibody, or proliferation of cancer cells (data not shown). 

To explore the importance of CCL5/CCR5 axis in the spread of tongue cancer, we performed a 3D invasion study with SAS and HSC-3 OTSCC cells with reduced CCR5 expression and with function-blocking antibody against CCL5. First, we inhibited the expression of CCR5 by transducing HSC-3 cells with CCR5-shRNA and produced a stable cell line with ~70 % inhibition of CCR5 expression and reduced response to rCCL5 induction (Figure 5C, 5D). In the 3D co-culture invasion assay the CCR5 knockdown cells invaded equally or than the wild-type or control HSC-3 cells transduced with non-silencing shRNA (Figure 5E, 5F). Similar results were also obtained with a monoclonal antibody against CCR5 (data not shown). 

### Expression of collagen and epithelial plasticity components are induced in cancer cells after BMMSC interaction

Potential mechanisms of the invasion promoting effect of BMMSCs on cancer cells were further studied by microarray analysis. HSC-3 cells were used for the experiments, since they responded more clearly to BMMSCs than SAS cells, especially in the 3D invasion model. HSC-3 cells were cultured as a monoculture or co-culture with BMMSC cells on 6-well plates for 24 h after which HSC-3 cells were sorted out from co-cultures with FACS (not shown) and RNA was extracted for microarray analysis. The overall gene profile of HSC-3 cells after interaction with BMMSCs showed up-regulated gene expression of ECM components, with the highest induction occurring for type I collagen mRNA expression. In addition to various collagens, BMMSC interaction with HSC-3 cells has also up-regulated modulators of ECM, such as lysyl oxidase (LOX) and other markers of epithelial plasticity, such as cadherin-11. Molecules associated with cell motility and cancer invasion, such as α-actinin-4 mRNAs, were also induced in HSC-3 cells by the interaction with BMMSCs (Table 3).

**Table 3:** Microarray analysis of up-regulated genes in HSC-3 cells after interacting with BMMSCs.

**Gene**	**Symbol**	**Fold Change**
collagen, type I, alpha 2	COL1A2	161.55
lumican	LUM	82.13
collagen, type I, alpha 1	COL1A1	67.11
periostin, osteoblast specific factor	POSTN	43.08
fibrillin 1 (Marfan syndrome)	FBN1	30.94
gremlin 1 homolog, cysteine knot superfamily (*Xenopus laevis*)	GREM1	30.83
collagen, type VI, alpha 3	COL6A3	30.28
platelet-derived growth factor receptor, beta polypeptide	PDGFRB	22.38
gap junction protein, alpha 1, 43kDa (connexin 43)	GJA1	21.12
collagen, type III, alpha 1 (Ehlers-Danlos syndrome type IV, autosomal dominant)	COL3A1	17.78
ABI gene family, member 3 (NESH) binding protein	ABI3BP	13.92
transgelin	TAGLN	5.61
chromosome 5 open reading frame 13	C5orf13	5.30
chondroitin sulfate proteoglycan 2 (versican)	CSPG2	4.38
serine (or cysteine) proteinase inhibitor, clade E (nexin, plasminogen activator inhibitor type 1), member 2	SERPINE2	4.26
angiopoietin-like 4	ANGPTL4	4.06
lysyl oxidase	LOX	3.67
collagen, type V, alpha 2	COL5A2	3.25
nicotinamide N-methyltransferase	NNMT	2.76
prostaglandin-endoperoxide synthase 2 (prostaglandin G/H synthase and cyclooxygenase)	PTGS1	2.47
heterogeneous nuclear ribonucleoprotein A1	HNRNPA1	2.41
cadherin 11, type 2, OB-cadherin (osteoblast)	CDH11	2.33
actinin, alpha 4	ACTN4	2.1

### Type I procollagen is highly expressed in the invasion front in tumor samples obtained from oral tongue cancer patients and correlated with worse prognosis

**Figure 6:**
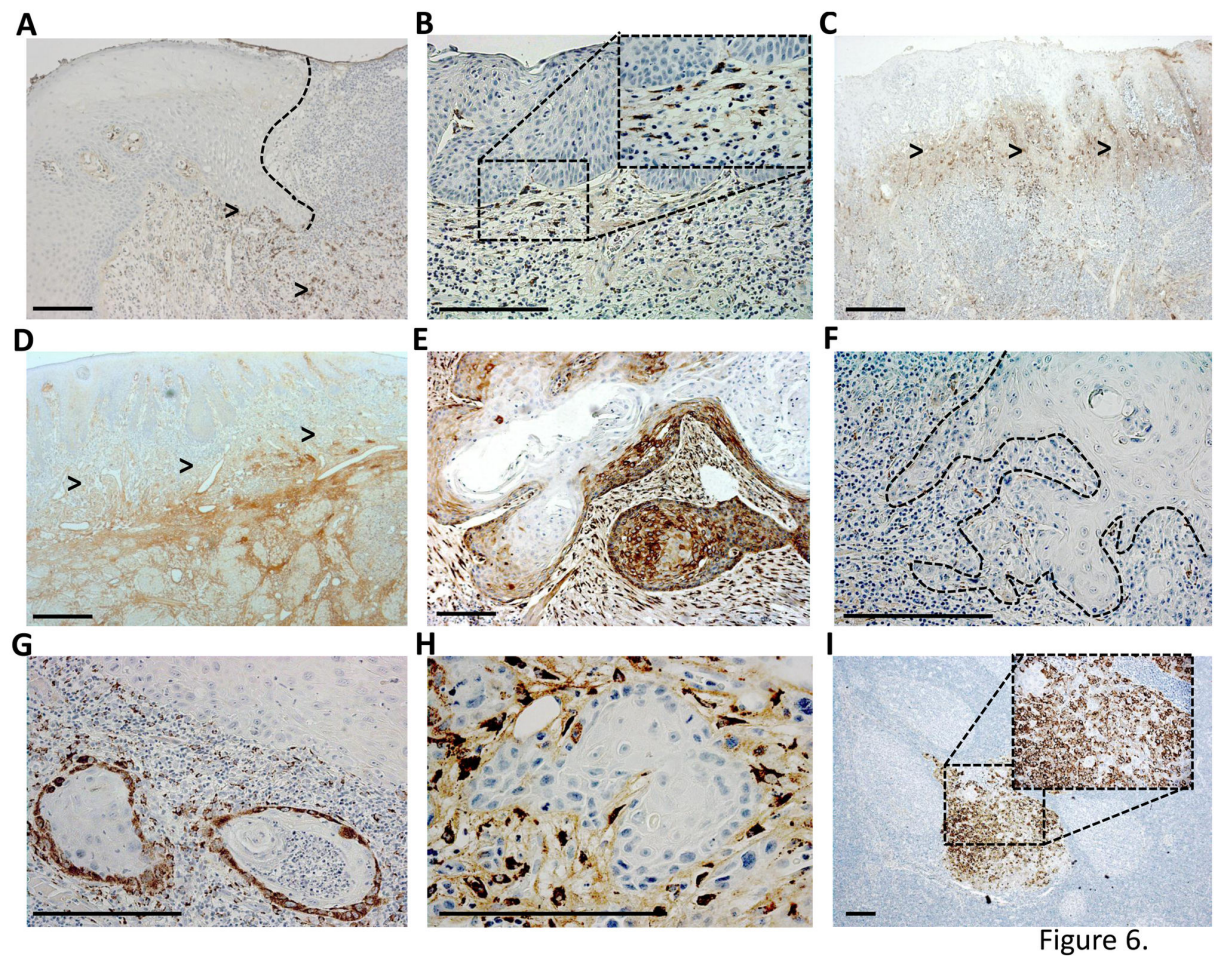
OTSCC samples show staining for type I collagen N-terminal propeptide (PINP). The expression of PINP was not found in any histologically normal epithelial cells of the carcinoma sections, except in a few of the mesenchymal cells within wound bed (arrow heads). The wound epithelial border is marked by dotted line (A). PINP expression was not found in dysplastic epithelium but was present in a few spindle cells in the submucosa (B). In various cases PINP expression was more intense in either superficial (C) or invasive (D) areas (arrow heads). Some cancers were mainly negative for PINP staining with a few sporatic positive areas (F), whereas other cases showed intense staining for both cancer and mesenchymal cells (E). In a couple of cases PINP was expressed in the periferal cells of invading islands (G) or mainly in the spindle carcinoma associated mesenchymal cells (H). Lymph node metastases were occasionally positive for PINP (I). Scale bar 200µm .

**Figure 7:**
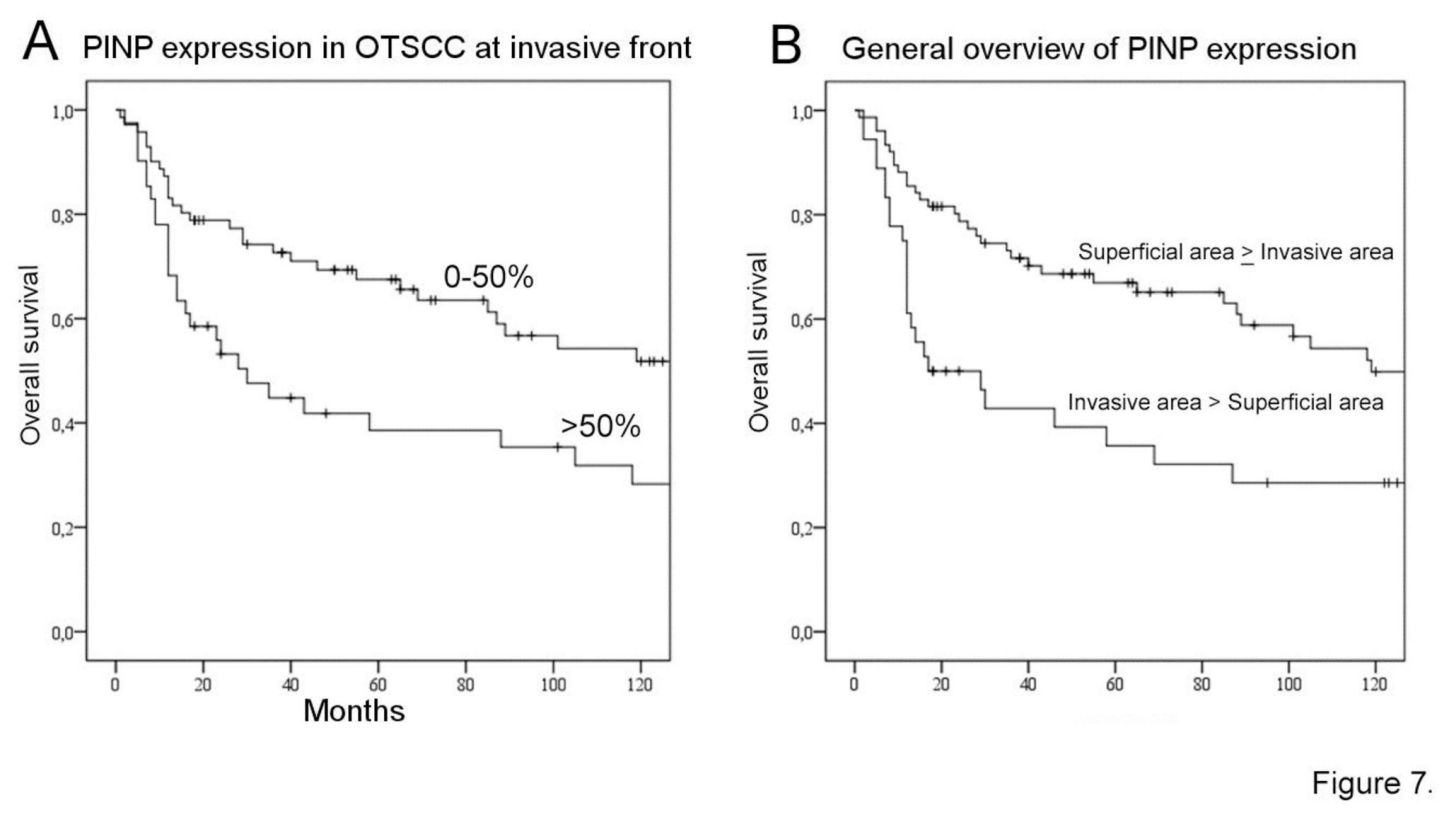
Survival analyses for PINP expression. In our set of samples, high PINP expression (PINP >50 %; n=41, 27 deaths in the group) compared to low expression (PINP 0-50%, n=71, 32 deaths) in OTSCC cells at the invasive front (>50%) correlated with increased overall deaths (A). In general overview, the increased expression of PINP in both OTSCC and stromal cells in the invasive areas of the tumor was observed in 76 patients (35 deaths in the group), whereas increased expression of PINP in superficial areas was observed in 36 patients (24 deaths). The higher expression of PINP in invasive areas associated with worse prognosis, p=0.004 (B).

To further evaluate the relevance of type collagen I expression originating from the cancer cell-stromal interactions to the aggressiveness of OTSCC, we performed immunohistochemistry for type I procollagen fragment (PINP). PINP has previously been shown to be associated with clinical outcome in breast and ovarian cancers, but so far has never been shown in OTSCCs [22-24,31]. In our 114 OTSCC cases, PINP was not found in epithelial cells in histologically normal nor dysplastic peritumoral tissue (Figure 6A-6B). In some tumors PINP was almost absent (Figure 6F), however, most of the cases showed high expression by both carcinoma and TME cells (Figure 6E, 6G, 6H). In general overview, PINP expression was more intense either in more invasive or superficial areas of the tumor (Figure 6C-D). Similar to the pattern of expression found in the primary tumors, PINP was also expressed in OTSCC lymph node metastases (Figure 6I). Furthermore, in a couple of lymph nodes originally signed out as free of metastases (pN0), PINP was expressed in a cluster of cells revealing previously unrecognized occult carcinoma metastases which also stained positive for pancytokeratin (not shown). We then compared the pattern of PINP expression and patient clinical data. In our survival analyses, there was a correlation between worse prognosis and expression of PINP by OTSCC cells at invasive areas (p=0.018) (Figure 7A). Increased PINP expression by both carcinoma and stromal cells at the invasive areas was also correlated to worse prognosis (p=0.004) (Figure 7B). Both the high expression of PINP in SCC cells (HR 1.728 95% CI [1.072 - 2.910]) as well as increased PINP expression by both carcinoma and stromal cells (HR 1.924, 95% CI [1.127 - 3.285]) at invasive areas were identified as independent indicators of overall survival along with patient age and high tumor stage. 

## Discussion

We studied here the role of bone marrow-derived BMMSCs in tongue cancer, which had not yet been previously addressed. We could demonstrate that BMMSCs enhanced the aggressiveness of OTSCC cells by inducing their invasion capacity, while reducing their proliferation levels. BMMSCs increased the expression of various chemokines, particularly CCL5, after interaction with HSC-3 cells. However, interference of CCL5 inhibited only the invasion area in our complex organotypic invasion assay. On the other hand, the interaction between cancer and bone marrow derived cells increased particularly the expression of type I collagen by carcinoma cells. The *in vivo* results derived from OTSCC patients´ samples showed that type I procollagen (PINP) antibody detected not only stromal mesenchymal cells, as expected, but interestingly also some of the OTSCC carcinoma cells, confirming our *in vitro* findings. Surrounding normal epithelial cells were negative for PINP staining. Importantly, the high expression of PINP in invasive areas of cancer correlated with the mortality of the patients. We speculate that the molecular cross-talk between BMMSCs and carcinoma cells leads to invasion promoting TME changes in OTSCC of which PINP, but not CCL5, could be used as prognostic marker for the OTSCC prognosis. In addition, the presence of PINP expression in cells within metastatic lymph nodes suggests the involvement of PINP also in the metastatic spread of the OTSCC. 

We wished to examine the invasion of aggressive tongue cancer cells using our 3D organotypic model [29] since this model represents a fully human, hypoxic tumor microenvironment [29], [41]. It is ideal for the studies of the early steps of invasion of tongue cancer, since it represents more faithfully the structure and composition of human ECM in tumors than the traditional invasion models, based on rat tail type I collagen and mouse EHS tumor derived Matrigel. We showed that in co-cultures, BMMSCs stimulated the dissemination of cancer cells and increased the depth of invasion, thus enhancing the aggressiveness of tongue cancer cells. Several studies have demonstrated the invasion and metastasis promoting role of BMMSCs in various cancers, such as breast, colon and lymphatic cancers [17], [18], [19]. However, the effect of BMMSCs on different tumor cells has remained controversial, potentially because of the different models and sources of BMMSCs used. Also, there may be differences in the responses of cancer cells and cancer subtypes to BMMSCs [20], [21]. In our experiments, we used two invasive tongue cancer cell lines and found that HSC-3, the most aggressive one, responded more efficiently to signals originating from BMMSCs in the 3D invasion model. However, in monolayer cell culture assays both OTSCC cell lines responded similarly to the BMMSC interactions. Differences in the responses in the 3D assays may partially be explained by different genetic background of these cells or by the transient effect of BMMSCs during the early steps of invasion similarly to breast cancer [17]. This transient effect is potentially difficult to see with the fairly aggressive cancer cells in the time-frame used in our 3D assays. In our study, BMMSCs were not able to induce an invasive phenotype in dysplastic DOK cells, suggesting differences in the capacity of invasive and non-invasive cells to respond to signals originating from BMMSCs. This also could reflect the EMT –process in which mesenchymal-like cells are more responsive to mesenchymal signaling. 

The roles of chemokines and their receptors in tongue cancer progression are not fully understood. Here we showed that BMMSCs increased the expression of various chemokines, including CCL5. This effect was more intense in the interaction between BMMSCs and tongue cancer cells than between normal fibroblasts and cancer cells. The neutralization of CCL5 activity with a function-blocking monoclonal antibody resulted in the inhibition of invasion area, but had no effect on invasion depth. This suggests that CCL5 might affect more the number of superficially disseminating invasive cells than the total invasive capacity of tongue carcinoma cells. In patient samples, however, the CCL5 expression was mainly detected in inflammatory cells in TME, some cancer cells and some sparse CAFs. BMMSCs have been shown to be one source of CAFs in TME [12,14]. Although we did not examine in detail the differentiation capacity of BMMSC, e.g. into CAFs when interacting with tongue cancer cells, our preliminary results from qt-RT-PCR analysis and immunostainings showed a clear increase in the gene expression of CAF-marker αSMA after the exposure of BMMSC to cancer cell culture media and more αSMA-positive BMMSCs in co-cultures with cancer cells than in BMMSC mono-culture (not shown). However, similarly to the results from other groups [20] we also detected the variability of the different batches of patient BMMSCs regarding the expression of αSMA, even though cell populations with low passage numbers were used. It remains to be shown if the effects seen in this study originate from BMMSCs or from BMMSCs differentiated to CAFs. 

The invasion and metastasis promoting effect of BMMSCs on breast cancer cells has been proposed to be essential and dependent on the CCL5-CCR5 axis [17,32,42]. In addition, antibodies against CCL5 have been shown to reduce the metastatic index in breast cancer [17] and CCR5 antagonists have been demonstrated to block metastasis of basal breast cancer cells [39]. CCL5/CCR5 axis has also been proposed to affect the motility of human osteosarcoma [40] and oral cancer cells *in vitro* [32] and CCL5 neutralization was shown to restrict tumor progression in colorectal carcinoma [33]. In tongue cancer the CCL5/CCR5 axis stimulated increased cell migration and the expression of MMP9 has been proposed to be mediated through NF-kappaB signaling pathways [32]. Although HSC-3 cells expressed CCR5 and recombinant CCL5 induced MMP9 expression through this receptor, the elimination of CCR5 activity with RNA interference or antibody against CCR5, and the reduction of MMP9 expression did not inhibit invasion in 3D invasion assay. MMP9 expressed here by this aggressive oral tongue HSC-3 cell line may only be of minor importance, since its expression was not significantly increased in cancer cells after BMMSC interaction based on the microarray analysis (Table 3). Instead, MMP in the BMMSCs [43] could be of importance in the regulation of the ECM proteins. The result also suggests compensatory utilization of other signaling pathways for CCL5, such as via CCR1 or CCR3 receptors, thus blocking only one signaling route may not be efficient enough to inhibit invasion in complex assays, such as 3D myoma organotypic model. 

The interaction with BMMSCs has particularly increased the expression of ECM proteins in tongue cancer cells. Interestingly, the highest induction occurred for type I collagen mRNA. Along with this finding, a paper has shown that fibrosis with type I collagen enrichment at the metastatic site can trigger the dormant-to-proliferative switch in dormant tumor cells leading to metastatic disease [44]. Recently, Shen and colleagues showed that fibrillar type I collagen is important in affecting the invasion of aggressive ovarian cancer cells [45] and Nguyen-Ngoc et al. demonstrated metastatic mammary tumors to preferentially disseminate in specific ECM microenvironment containing also type I collagen [46]. More interestingly, transcriptomic dissection of OTSCC patient samples has revealed enhancements in e.g. ECM organization and biogenesis, and collagen catabolism. Type I collagen was one of the up-regulated genes in the signature gene sets for OTSCC [47]. In addition, modulation of molecules associated to cell motility and cancer invasion, for example, the down-regulation of α-actinin-4, has recently been shown to decrease invasion potential in oral squamous cell carcinoma [48]. Although in our OTSCC cells there were no remarkable changes in the gene expression of traditional EMT markers [49], after the interaction with BMMSCs, up-regulation in the expression of EMT plasticity biomarkers, such as cadherin-11, was detected. We speculate that the increase in the expression of all these components may indicate invasion promoting changes in the extracellular vicinity of cancer cells. However, more studies are needed to clarify these observations at cellular level.

Our OTSCC patient samples showed that PINP antigen, representing the procollagen fragment of type I collagen, was present not only in stromal cells, as expected, but also in OTSCC carcinoma cells and in lymph node metastases. Thus, the results supported the above mentioned *in vitro* observation demonstrating the increase of type I collagen expression in cancer cells that interact with stromal cells, including BMMSCs. So far, PINP expression has not been described in OTSCCs. However, the fibroproliferative TME response, or desmoplastic reaction, has already been reported as a prognostic predictor of occult metastasis in OTSCCs [50]. The similarity between desmoplastic reactions in cancer stroma and wound healing are evident in type I collagen processes [51]. PINP has been demonstrated to be a prognostic marker in primary and secondary brain tumors [52] and breast cancer [52] as well as osteosarcoma cells, osteoblasts and proliferating fibroblasts [53,54]. Our results proved that the high PINP expression at the invasive front serves as a reliable indicator for aggressive growth and metastases and could be useful as a prognostic marker also in OTSCCs.

To conclude, BMMSCs promoted the invasion of tongue carcinoma cells by inducing the expression of components associated to chemokine signaling, epithelial plasticity, cell motility and invasion, potentially promoting invasion favoring changes in cancer cells and in the TME. Although CCL5 expression was induced in BMMSCs after interaction with OTSCC cells, signaling through CCL5/CCR5 axis does not seem to be critical for the BMMSC enhanced cancer invasion. Instead, the induction of ECM protein production, particularly type I collagen, by cancer cells after BMMSC interaction turned out to be more crucial for OTSCC behavior *in vitro*. Moreover, based on our clinical data we suggest that PINP antibody, recognizing type I collagen N-terminal propeptide, is an appropriate prognostic marker for OTSCC.
