# Correction: Human Bone Marrow Mesenchymal Stem Cells Induce Collagen Production and Tongue Cancer Invasion

**DOI:** 10.1371/annotation/2bacf09d-7bb8-43ac-a098-b3b9e486d854

**Published:** 2013-11-15

**Authors:** Sirpa Salo, Carolina Bitu, Kalle Merkku, Pia Nyberg, Ibrahim O. Bello, Jussi Vuoristo, Meeri Sutinen, Hannu Vähänikkilä, Daniela E. Costea, Joonas H. Kauppila, Petri Lehenkari, Dan Dayan, Marilena Vered, Juha Risteli, Tuula Salo

The name of the 10th author was incorrectly given. The correct name is: Joonas H. Kauppila. The correct abbreviation in the Author Contributions Statement is: JHK. 

